# Successful Hysteroscopic Removal of a Retained Karman Cannula Tip: A Case Report

**DOI:** 10.7759/cureus.93930

**Published:** 2025-10-06

**Authors:** Pradip Kumar Saha, Anju Singh, Mamta K Chaudhary, Rashmi Bagga, Anika Saraf

**Affiliations:** 1 Obstetrics and Gynaecology, Postgraduate Institute of Medical Education and Research, Chandigarh, IND

**Keywords:** broken karman cannula, hysteroscopic foreign body retrieval, retained foreign body, standard operating procedure (sop) for suction evacuation, suction evacuation complication

## Abstract

The most frequently performed procedure for first-trimester abortions is suction evacuation due to its excellent safety profile. Complications of suction evacuation include bleeding, infections, sepsis, retained products, and, very rarely, retention of the broken tip of the Karman cannula. The literature on the retention of a broken Karman cannula tip is very scarce. We present a case of a retained Karman cannula tip during suction evacuation for a medical termination of pregnancy in a 37-year-old woman with G2P1L1 and a previous cesarean section at 12 weeks of gestation. Ultrasonography (USG)-guided retrieval with forceps was attempted but was unsuccessful. The patient was managed with hysteroscopic removal of the broken Karman cannula tip using hysteroscopic polyp forceps. The purpose of this case report is to highlight this rare but challenging complication, document the management method, and discuss points to consider during suction evacuation to prevent such occurrences.

## Introduction

The safest and most preferred method for first-trimester medical termination of pregnancy is suction evacuation [1]. Complications are uncommon and primarily include hemorrhage, uterine or cervical trauma, and infection. This procedure uses a plastic cannula designed by Karman approximately 50 years ago [2]. However, breakage and retention of the cannula during suction evacuation are rarely documented in the literature. Hysteroscopy-guided removal of uterine foreign bodies is known to reduce the risk of collateral damage during the procedure. One of the earliest reports of hysteroscopic removal of a broken Karman cannula was published in the late 1970s by Damadia et al. [3], but such cases remain infrequent. We aim to contribute to the literature on this rare occurrence, including management strategies for this challenging situation and methods to prevent it.

## Case presentation

A 37-year-old woman, gravida 2, para 1, live 1 (G2P1L1), with a previous cesarean section, presented at 10 weeks of gestation for medical termination of pregnancy due to maternal dilated cardiomyopathy with an ejection fraction of 20%, as the patient was concerned about the high perinatal mortality rate related to her comorbidities. Her gestational age was 10 weeks, calculated from her last menstrual period and confirmed by ultrasonography.

She has been a known case of diabetes, managed with metformin and dapagliflozin for the past year. She was diagnosed with dilated cardiomyopathy two years ago and was on medications, including torsemide, spironolactone, metoprolol, and telmisartan. During examination, her vital signs were within normal limits, with a NYHA class I and a BMI of 35.

On examination, the abdomen was soft, with a previous Pfannenstiel scar. Per vaginam, the cervix was firm, the uterus was posterior, approximately 10 weeks' gestation, and the external os was closed. She was scheduled for medical termination of pregnancy via surgical method due to her medical comorbidities.

The patient was scheduled for USG-guided suction evacuation with a Karman cannula number 10 and Levonorgestrel intrauterine device insertion under anesthesia. During the procedure, slight resistance was noted at the level of the internal os while removing the cannula. The tip of the Karman cannula was missing, and the patient experienced active bleeding. Multiple unsuccessful attempts were made to retrieve the tip using retrieval forceps. A decision was made to perform hysteroscopy-guided removal.

**Figure 1:**
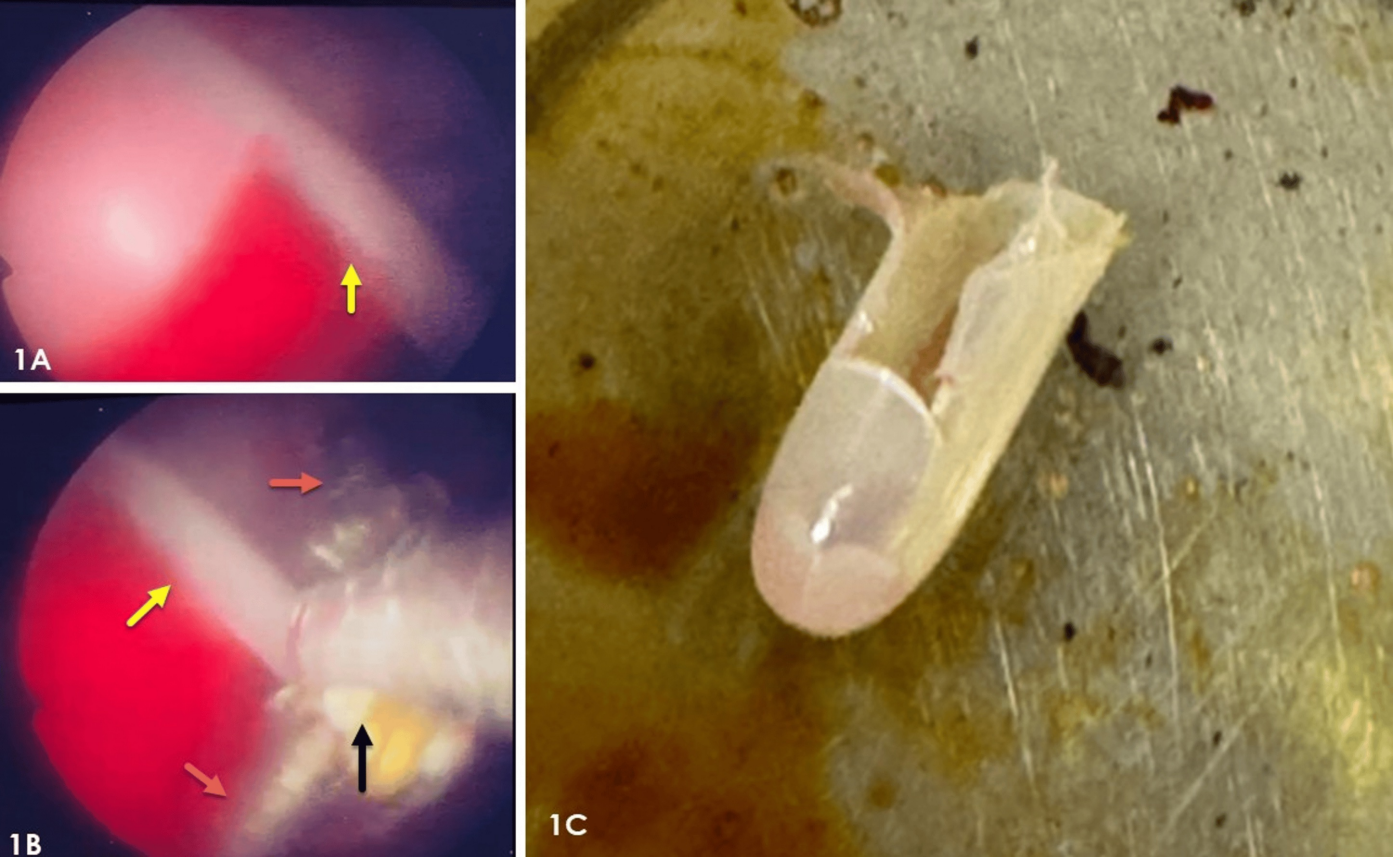
Clinical photographs showing hysteroscopic view during the retrieval procedure and the broken tip after retrieval. 1A: Hysteroscopic view of retained tip of Karman cannula (yellow arrow). 1B: Hysteroscopic view showing hysteroscopic polyp forceps body (black arrow) and arms (orange arrows) holding one edge of the retained tip (yellow arrow). 1C: Photograph of the broken tip of the Karman cannula after retrieval.

During hysteroscopic examination, visualization was challenging due to heavy bleeding. Nevertheless, the tip of the Karman cannula with products of conception in place was ultimately seen on the left lateral wall of the mid-uterus segment (Figure 1A).

The cannula tip was grasped with hysteroscopic polyp forceps (Figure 1B) in a longitudinal orientation to align with the cervical canal. It was then brought to the cervical canal under hysteroscopic visualization by hysteroscopic polyp forceps and finally removed using artery forceps (Figure 1C). After the procedure, the uterine cavity was empty. The postoperative period was uneventful, and the patient was advised to follow up in six weeks for contraception counselling. An intrauterine device for contraception was inserted 15 days after the evacuation.

## Discussion

This case highlights the unexpected challenges that can catch a surgeon off guard during a procedure usually considered safe. The retention of the Karman tip during the procedure is an obstetrician's nightmare. With only a few reports available, the literature remains scarce regarding the retention of a broken Karman cannula during suction evacuation. Diagnosis may be delayed because of trapped fragments in the myometrium. USG can assist in identifying the nature of a foreign body [4]. The hysteroscopic approach remains the primary method for retrieving these foreign bodies because of its clear advantage of direct visualization. The success of the hysteroscopic approach depends on the surgeon's expertise and the position of the foreign body. Yazicioglu et al. described a case where the tip of the Karman cannula was missed during endometrial sampling, leading to a perforation below the internal os in the anterior wall of the cervix, which ultimately caused the missed cannula tip to reach the subvesical retroperitoneal space [5]. Such a difficult location required additional attempts during retrieval. However, it was successfully completed under hysteroscopic guidance. Having a backup plan for an emergency laparotomy in such cases can be extremely helpful.

In our case, attempts to remove the cannula tip with artery forceps under USG guidance were unsuccessful, and the patient began bleeding heavily, making it even more difficult to locate the foreign body. Bleeding, along with infection, uterine perforation, and migration through the perforation track, are common adverse outcomes. Prolonged retention due to misdiagnosis is also a reported sequela [6].

The measures aimed at preventing breakage and retention of the cannula tip involve selecting an appropriately sized curette. The curette is inserted into the uterine cavity while stabilizing the cervix with a sponge holder. The suction hose may be attached either prior to or following the insertion of the curette, with the suction valve kept turned off initially. It is advisable to keep the suction valve closed until the optimal suction level is established. Once the suction is activated, the curette may be rotated 360 degrees continuously until no further material is aspirated. Subsequently, the suction curette is withdrawn with the suction valve remaining closed [7]. Establishing a standardized operating procedure for counting and inspecting all instruments before and after the procedure can aid in early detection of any retained foreign bodies. Recent developments include the utilization of a radio-opaque tip on a Karman cannula or mini-chips for radiofrequency identification, which are affixed to the instruments [8]. In addition, performing routine ultrasound examinations prior to patient discharge can help in ruling out any retained foreign bodies.

## Conclusions

The key approach in managing a retained Karman cannula involves early diagnosis and removal through hysteroscopy with direct visualization, followed by an ultrasound to ensure the uterine cavity is empty. Preventive measures include pre- and post-procedure audits of all instruments and careful handling of the curettes. Moreover, advances in tracking retained fragments, such as radio-opaque materials and radiofrequency identification, should be incorporated during the procedure.
